# Adolescent sleep characteristics and body-mass index in the Family Life, Activity, Sun, Health, and Eating (FLASHE) Study

**DOI:** 10.1038/s41598-020-70193-w

**Published:** 2020-08-06

**Authors:** Aaron C. Schneider, Dong Zhang, Qian Xiao

**Affiliations:** 1 Department of Health and Human Physiology, University of Iowa, Iowa City, IA 52242 USA; 2 Department of Epidemiology, Human Genetics, Environmental Sciences, The University of Texas Health Science Center at Houston, Houston, USA

**Keywords:** Weight management, Risk factors

## Abstract

Sleep may play a role in overweight and obesity in adolescents. The objective of this study is to investigate the relationships between sleep duration and timing and overweight and obesity status in adolescents, with a special emphasis on weekday–weekend difference in sleep characteristics as well as sex-specific relationships. We examined 1,254 U.S. adolescents (12–17 years) self-reported sleep duration, timing, weekday–weekend differences in duration and timing in relation to overweight and obesity. We found an inverse association between sleep duration and overweight and obesity. Compared to 8–9 h of sleep, short sleep (< 7 h) on weekdays was associated with higher odds of overweight and obesity [Odds ratio (95% confidence interval), 1.73 (1.00, 2.97)] in the overall population, while long sleep (10+ h) on weekends was associated with lower odds, but only in males [0.56 (0.34, 0.92)]. We also found that a larger weekday–weekend difference in sleep duration was associated with overweight and obesity in females, but not in males. Specifically, the odds of overweight and obesity were significantly higher among females reporting longer sleep on weekends than weekdays by ≥ 2 h [2.31 (1.15, 4.63)] when compared to those reporting little weekday–weekend differences. Sleep timing, or weekday–weekend differences in sleep timing, were not associated with overweight and obesity in the overall population, although we found suggestive evidence linking later weekend sleep with overweight and obesity in females. Our findings support a role of sleep in adolescent obesity and suggest sex-differences in this relationship that warrant future studies.

## Introduction

Adolescents who are overweight and obese are at a greater risk of developing a wide range of health conditions in adulthood, including high blood pressure, heart disease and strokes, certain types of cancer, kidney disease, and type II diabetes^1^. Emerging evidence suggests that sleep deficiency may be an important risk factor for overweight and obesity in childhood and adolescence. However, findings from previous studies are mixed, with some reporting negative associations between sleep duration and body-mass index (BMI)^2–5^, others reporting U-shaped associations similar to findings in adults^6^, and some reporting no associations at all^7^.

Recent studies suggested that later sleep timing and propensity of eveningness may also be associated with higher BMI^8–12^. This may be due to more severe misalignment between the internal circadian cycles and the behavioral sleep–wake cycles that are partially influenced by social and environmental factors such as school schedules and social obligations^13,14^. It has been well documented that chronotype shifts later during adolescent years^14^. Many middle and high schools have a relatively early start time, and this mismatch between school schedules and chronotype-determined sleep window may lead to sleep deficiency on weekdays among adolescents. Moreover, sex-differences in chronotype and sleep patterns have been reported among adolescents, and it has been suggested that both biological and behavioral factors may contribute to such differences^13,15,16^. Therefore it is particularly important to examine the relationship between sleep and overweight and obesity and explore potential sex-differences in sleep–BMI relationship in adolescent populations.

In addition to average sleep duration and timing, greater weekday–weekend variability in sleep patterns may also play a role in overweight and obesity. Several studies found that adolescents who reported sleeping longer on weekends relative to weekdays have a lower BMI^17,18^, although other studies linked a greater weekday–weekend difference in sleep timing, also known as the social jet lag, to obesity^11,13,19^. However, studies focusing on weekday–weekend sleep variabilities in adolescents are still limited.

In a group of ~ 1,200 US adolescents aged 12–17 years old, we examined the cross-sectional relationship between sleep duration and timing and overweight and obesity status, with a special emphasis on weekday–weekend difference in sleep characteristics as well as sex-specific relationships between sleep and BMI. Our research hypothesized, sleeping outside of optimal biological timing and duration, as well as compensating on weekends due to weekday sleep loss, would be associated with higher BMI. Specifically, shorter sleep duration, later sleep timing, and greater weekday–weekend differences in sleep duration and timing would be associated with overweight and obesity in this population.

## Methods

### Study population

We conducted our analysis using data from the National Cancer Institute’s Family Life, Activity, Sun, Health, and Eating (FLASHE) Study. FLASHE is a cross-sectional survey administered to dyads of parents and their adolescent child (aged 12–17) between April and October 2014. Details of the FLASHE study design and measures development have been published before^20^. Parent participants were recruited to the study through the Ipsos Consumer Opinion Panel, a national market research firm, and were eligible if they were aged ≥ 18 years and lived with at least one child aged 12–17 years for at least 50% of the time. During screening, one eligible adolescent from the household was randomly selected for the study. Informed consent was obtained from parents or legal guardians, and on behalf of their adolescent under their care. Consent was acquired via study website which required contact information, consent (parent consent and parent consent for adolescent), and assent (adolescent) to be fully enrolled. The invitations described the study procedures, incentives, and contacts. Consent/assent forms described data privacy and that participation was voluntary, and indicated that both dyad members were required to enroll. FLASHE was reviewed and approved by the U.S. Government’s Office of Management and Budget, the National Cancer Institute Special Studies Institutional Review Board (IRB), and the Westat IRB^21^.

Among the 1,737 adolescents in FLASHE, we excluded adolescents with missing data on demographics and sleep variables to calculate duration, midpoints, or social jet lag, resulting in a final analytic sample of 1,254 adolescents, including 622 males and 632 females. Invitations were sent to parents’ and adolescents’ e-mail addresses and contained the URL for the study website and a personalized identification number.

### Adiposity

In this study, BMI was calculated from the self-reported height and weight of the adolescent participants and categorized into underweight (< 5th percentile), healthy weight (5th to < 85th percentile), overweight (85th to < 95th percentile), and obese (≥ 95th percentile) groups based on BMI-z scores obtained from the Center for Disease Control (CDC) recommended BMI-for-age Cutoffs^22^. There is high correlation between adolescent BMI calculated from self-report and measured height and weight^23–25^ and self-reported height and weight have been validated to have high agreement with measured values^26^. The primary outcome for this study was overweight/obesity defined as BMI > 85th percentile for age derived from the CDC cutoffs.

### Sleep assessment

Self-reported time to bed and time out of bed (hour, minute, AM or PM) were obtained for both school nights (Sunday–Thursday) and weekend nights (Friday and Saturday). From these we calculated weekday sleep duration, weekday midpoint, weekend duration, weekend midpoint, and weekday–weekend differences in midpoint and duration. Sleep duration for weekdays and weekends was categorized as < 7 h, 7–8 h, 8–9 h, 9–10 h, and 10+ h with the largest category (8–9 h) chosen as the referent group. Weekday–weekend difference in sleep duration was divided into quintiles (< 0, 0 to < 0.5 h, 0.5 to < 1.33 h, 1.33 to < 2 h, and ≥ 2 h), and the second quintile was used as the reference as it represents the smallest absolute difference between weekdays and weekends. Midpoint of sleep on weekdays and weekends as well as the weekday–weekend difference in midpoint were categorized into quintiles, and we used the 1st quintile for each variable as the referent group, because previous literature suggested that midpoint and social jet lag were inversely associated with BMI^27^.

### Covariates

Physical activity was measured by the Youth Activity Profile^28^ survey, which asks about activity patterns both during and out of school the previous week, and we derived the total minutes of moderate-to-vigorous physical activity (MVPA) during school, out of school, and on weekends using a calibration model reported before^29^. FLASHE also collected information on sociodemographic factors of the children and their parents, as well as diet and TV viewing behaviors. A 27-item dietary screener was administered among parents and adolescents, which assessed the daily frequency of intake for each food item, ranging from “never” to “3 or more times per day”. We used the summed daily frequencies of all “detrimental” food and “beneficial” food as the measures of dietary quality. Examples of detrimental food include pizza, fried foods, burgers, processed meat, candy/chocolate, and examples of beneficial food include water, fruit, green salad, non-fried vegetables, and whole grain bread.

### Statistical analysis

Descriptive statistics are shown as means and standard deviations for continuous variables, and frequencies and percentages for categorical variables. Odds ratios (OR) and 95% confidence intervals (CI) were estimated using logistic regression models using SAS 9.4 (SAS Institute, Cary, NC, USA). We included multiple variables as potential confounders in a series of models: the base model was adjusted for sex (not adjusted in sex-specific analysis) and age; model 2, which we considered our main model, was additionally adjusted for other potential confounders including race (Hispanic, Black or African American only, white only, or other), type of school (private, public, home schooled, or other), parental marital status (married, divorced widowed or separated, never married, member of unmarried couple), homeownership (own/not own), and parental education (less than a high school degree, high school degree or GED, some college but not a degree, 4-year college degree or higher); in model 3 we additionally included variables that could be both confounders and mediators, including daily frequency of detrimental food, daily frequency of beneficial food, MVPA, and time spent watching television (0, less than 1 h, 1–2 h/day, more than 3 h/day), as previous literature has shown a bi-directional relationship between sleep and physical activity and diet^30,31^. To test for trend, we assigned a numerical value (1 through 5) to each category of sleep variables.

## Results

Table 1 presents baseline characteristics of participants according to weekday sleep duration. Compared with adolescents in the 8–9 h sleep category, those with shorter sleep were older and less likely to be white, reported less MVPA, had parents with higher education, ate more servings of beneficial food but less detrimental food, watched more television, and had a higher BMI. On the other hand, those who reported longer sleep were younger, less likely to be in a public school, ate more beneficial food, and reported higher MVPA and less TV viewing.

**Table 1:** Baseline characteristics from the Family Life, Activity, Sun, Health, and Eating (FLASHE) Study.

Weekday sleep duration, hours/day					
	< 7 (n = 75)	7–8 (n = 247)	8–9 (n = 411)	9–10 (n = 357)	10+ (n = 164)
**Gender (%)**					
Female	49.33	50.61	52.55	46.78	52.44
Male	50.67	49.39	47.45	53.22	47.56
**Age, mean (SD), years**	15.28 (1.41)	15.19 (1.32)	14.64 (1.54)	13.92 (1.61)	13.77 (1.55)
**Race/ethnicity (%)**					
Hispanic	12.00	9.31	8.52	8.96	10.37
Black or African American	21.33	20.24	15.57	14.01	12.20
White only	53.33	61.94	65.69	67.51	66.46
Other	12.00	8.10	8.76	8.96	9.76
**School type (%)**					
Public	90.67	88.66	88.81	80.39	69.51
Private	6.67	8.50	6.81	9.80	4.27
Home-schooled	2.67	1.21	2.92	6.16	23.17
Other	0.00	1.62	1.46	3.08	3.05
**Parental marital status (%)**					
Married	72.00	70.85	71.53	72.83	75.61
Divorced-widowed or separated	14.67	13.77	12.65	10.08	9.15
Never married	6.67	9.72	7.06	9.24	8.54
Member of unmarried couple	5.33	4.05	7.06	5.88	3.66
**Home ownership (%)**					
Own	73.33	73.28	72.75	72.27	68.29
Not own	25.33	24.7	25.55	25.77	29.88
**Parental education (%)**					
Less than high school degree	0.00	1.21	0.73	1.40	2.44
High school degree or GED	16.00	15.38	17.76	13.45	18.90
Some college but no degree	26.67	31.98	32.60	36.69	37.20
4-year college degree or higher	54.67	51.01	47.45	46.78	40.85
**Daily frequency of detrimental food (servings) (%)**	2.77 (7.95)	3.53 (4.63)	3.78 (4.68)	3.42 (6.08)	3.64 (5.63)
**Daily frequency of beneficial food (servings) (%)**	5.71 (5.36)	4.21 (4.24)	4.44 (4.8)	4.89 (5.16)	5.28 (4.76)
**MVPA, mean (SD), min/week (%)**	613.8 (125.42)	625.52 (109.92)	667.29 (117.4)	717.24 (117.03)	733.01 (124.19)
**Television time (%)**					
Really don't watch	10.67	6.88	5.60	6.72	9.76
< 1 h	20.00	16.19	17.27	19.89	18.90
1–2 h	25.33	31.58	36.74	36.41	23.17
2–3 h	13.33	21.86	24.57	16.53	23.78
> 3 h	18.67	18.22	12.41	11.48	6.71
**BMI (%)**					
Underweight	5.33	3.24	4.62	3.92	7.32
Healthy weight	60.00	71.26	69.34	69.19	70.12
Overweight	16.00	13.77	15.33	14.85	9.76
Obese	18.67	11.74	10.71	12.04	12.80

*BMI* body mass index [weight (kg)/height (m^2^)], *GED* general education development, *MVPA* moderate-vigorous physical activity, *SD* standard deviation.

Because of missing values for some categories total number may not add up to 1,254 or 100%

In Table 2 we present the associations between sleep duration and being overweight or obese. In the overall population, we found an inverse association between sleep duration and overweight and obesity, particularly for weekday sleep (p-for-trend, 0.03). Specifically, short sleep (< 7 h) on weekdays was associated with a higher likelihood of being overweight or obese [OR (95% CI) 1.73 (1.00, 2.97)], when compared to the reference group (8–9 h). The association was attenuated after including potential mediators, MVPA, diet, and TV time [1.57 (0.82, 3.01)]. This association was similar in both males and females, although the linear trend was only statistically significant in males. For weekend sleep duration, we found a significant trend between longer sleep and higher odds of overweight and obesity, but only in males (p-for-trend, 0.01). Moreover, this association appeared to be primarily driven by 10+ h of sleep, which was associated with 44% decrease in the odds of overweight and obesity [0.56 (0.34, 0.92)] in males. Adjusting for MVPA, diet and TV time had minimal impact on the association between long sleep and overweight and obesity in males [0.52 (0.31, 0.88)]. In contrast, short sleep duration on weekends was not associated with overweight and obesity in the overall population or in sex-specific analysis.

**Table 2:** Associations between weekday and weekend sleep duration and overweight and obesity among adolescents (age 12–17, N = 1,254).

	Sleep duration, hours										*p* for Trend
	< 7		7–8		8–9		9–10		10+		
	Weekday										
	OR 95% CI		OR 95% CI		OR 95% CI		OR 95% CI		OR 95% CI		
**Overall**											
Base model	1.63	(0.96, 2.77)	1.06	(0.73, 1.52)	Referent		0.91	(0.65, 1.27)	0.72	(0.46, 1.11)	0.02
Model 2	1.73	(1.00, 2.97)	1.05	(0.72, 1.53)	Referent		0.94	(0.67, 1.31)	0.73	(0.46, 1.17)	0.03
Model 3	1.57	(0.82, 3.01)	0.94	(0.61, 1.45)	Referent		0.98	(0.65, 1.46)	0.59	(0.32, 1.07)	0.06
**Females**											
Base model	1.50	(0.69, 3.29)	1.17	(0.69, 1.99)	Referent		1.10	(0.67, 1.78)	0.90	(0.48, 1.67)	0.35
Model 2	1.81	(0.80, 4.07)	1.20	(0.70, 2.07)	Referent		1.11	(0.68, 1.84)	0.97	(0.50, 1.86)	0.34
Model 3	1.84	(0.72, 4.70)	1.02	(0.52, 1.97)	Referent		1.25	(0.68, 2.31)	0.98	(0.42, 2.30)	0.64
**Males**											
Base Model	1.69	(0.81, 3.54)	0.94	(0.57, 1.57)	Referent		0.77	(0.49, 1.22)	0.56	(0.30, 1.06)	0.02
Model 2	1.65	(0.77, 3.52)	0.94	(0.56, 1.60)	Referent		0.80	(0.49, 1.27)	0.54	(0.27, 1.08)	0.03
Model 3	1.60	(0.71, 3.58)	0.86	(0.50, 1.48)	Referent		0.76	(0.46, 1.24)	0.52	(0.25, 1.06)	0.04
	Weekend										
**Overall**											
Base model	1.35	(0.65, 2.78)	1.03	(0.58, 1.84)	Referent		0.93	(0.62, 1.37)	0.80	(0.55, 1.15)	0.06
Model 2	1.23	(0.58, 2.58)	1.08	(0.60, 1.95)	Referent		0.94	(0.63, 1.41)	0.78	(0.54, 1.14)	0.06
Model 3	1.03	(0.38, 2.84)	0.98	(0.48, 1.99)	Referent		0.91	(0.56, 1.48)	0.85	(0.56, 1.33)	0.03
**Females**											
Base model	1.90	(0.57, 6.32)	1.16	(0.48, 2.81)	Referent		1.05	(0.55, 2.00)	1.23	(0.68, 2.21)	0.94
Model 2	1.62	(0.46, 5.69)	1.13	(0.45, 2.83)	Referent		0.95	(0.48, 1.86)	1.07	(0.58, 1.98)	0.79
Model 3	1.60	(0.28, 9.17)	1.01	(0.30, 3.36)	Referent		1.01	(0.44, 2.32)	1.15	(0.53, 2.50)	0.71
**Males**											
Base model	1.18	(0.47, 2.97)	1.02	(0.47, 2.25)	Referent		0.90	(0.54, 1.49)	0.57	(0.35, 0.93)	0.01
Model 2	1.12	(0.44, 2.89)	1.09	(0.48, 2.45)	Referent		0.93	(0.55, 1.56)	0.56	(0.34, 0.92)	0.01
Model 3	1.03	(0.38, 2.78)	1.07	(0.46, 2.48)	Referent		0.92	(0.53, 1.57)	0.52	(0.31, 0.88)	0.01

Weekday consists of sunday to thursday evenings. Weekend consists of Friday and saturday evenings.

Overweight-obese defined as body mass index > 85th percentile.

Base Model adjusted for sex (for overall analysis alone) and age.

Model 2: adjusted for covariates in base model and race, school type, parental marital status, home ownership, parental education.

Model 3: adjusted for covariates in Model 2 and moderate-vigorous physical activity (MVPA), teen sedentary time, teen frequency of beneficial foods eaten, teen frequency of detrimental foods eaten, and time spent watching television.

p-for-interaction between sex and sleep duration: 0.78 for weekday and 0.15 for weekend sleep duration.

*OR* Odds ratio, *95% CI* 95% confidence interval.

For weekday–weekend difference in sleep duration, we found that in the overall population, a larger weekday–weekend difference was associated with higher odds of being overweight or obese, when compared to the reference group with the minimal difference between weekday and weekend sleep duration (Table 3). Specifically, those who reported shorter sleep on weekends had an 81% increase in the odds of being overweight or obese [1.81 (1.13, 2.91)]. Moreover, those who reported a longer weekend sleep by 2 h or more demonstrated a 60% increase [1.60 (1.02, 2.51)], and this association was particularly striking in females [2.31 (1.15, 4.63)], although the interaction term between weekday–weekend difference in sleep duration and sex was only borderline significant (p = 0.07). When we additionally adjusted for average sleep duration on both weekdays and weekends, we observed little impact on the associations (data not shown).

**Table 3:** Associations between weekday and weekend difference in sleep durations and overweight and obesity among adolescents (age 12–17, N = 1,254).

Range of weekend–weekday difference in sleep duration, hours	Weekend–weekday difference in sleep duration, quintiles									
	− 6 to 0		0.00 to 0.67	0.67 to 1.50		1.51 to 2.00		2.01 to 5.00+		*p* for Trend
	OR 95% CI		OR 95% CI	OR 95% CI		OR 95% CI		OR 95% CI		
**Overall**										
Base model	1.79	(1.13, 2.83)	Referent	1.25	(0.80, 1.95)	1.40	(0.86, 2.27)	1.67	(1.08, 2.60)	0.67
Model 2	1.81	(1.13, 2.91)	Referent	1.26	(0.80, 1.99)	1.38	(0.84, 2.26)	1.60	(1.02, 2.51)	0.89
Model 3	2.08	(1.12, 3.88)	Referent	1.84	(1.01, 3.33)	1.50	(0.79, 2.84)	1.59	(1.18, 3.80)	0.89
**Female**										
Base model	2.10	(0.98, 4.50)	Referent	1.68	(0.83, 3.38)	1.58	(0.74, 3.33)	2.45	(1.25, 4.79)	0.13
Model 2	2.15	(0.97, 4.77)	Referent	1.74	(0.84, 3.63)	1.46	(0.67, 3.20)	2.31	(1.15, 4.63)	0.26
Model 3	2.15	(0.75, 6.19)	Referent	1.98	(0.76, 5.14)	1.25	(0.45, 3.47)	2.59	(1.04, 6.45)	0.36
**Male**										
Base model	1.60	(0.89, 2.89)	Referent	1.01	(0.56, 1.80)	1.37	(0.72, 2.64)	1.17	(0.64, 2.16)	0.43
Model 2	1.56	(0.85, 2.86)	Referent	0.99	(0.54, 1.81)	1.28	(0.65, 2.52)	1.02	(0.54, 1.92)	0.24
Model 3	1.46	(0.78, 2.74)	Referent	1.00	(0.53, 1.86)	1.19	(0.59, 2.40)	0.93	(0.48, 1.79)	0.20

Weekday consists of sunday to thursday evenings. Weekend consists of friday and saturday evenings.

Base model adjusted for sex (for overall analysis alone) and age.

Model 2: adjusted for covariates in base model and race, school type, parental marital status, home ownership, parental education.

Model 3: adjusted for covariates in model 2 and moderate-vigorous physical activity (MVPA), teen sedentary time, teen frequency of beneficial foods eaten, teen frequency of detrimental foods eaten, and time spent watching television.

Negative weekend–weekday difference in duration values (< 0) represent longer sleep on the weekdays compared to weekends.

Positive weekend–weekday difference induration values (> 0) represent longer sleep on the weekend compared to weekdays.

p-for-interaction between sex and weekday–weekend difference in sleep duration: 0.07.

*OR* Odds ratio, *95% CI* 95% confidence interval.

Next we examined the relationship between midpoint of sleep and overweight and obesity (Table 4). Neither weekday nor weekend midpoint were associated with overweight and obesity in the overall population or in male participants. However, there appeared to be a trend in females suggesting a relationship between a later midpoint on weekends and higher odds of overweight and obesity (p-for-trend, 0.02), with those who reported a weekend midpoint after 4 a.m. showing ~ 80% increase in overweight and obese when compared to the reference group (early than 3:30 a.m.). Interaction between weekend midpoint and sex demonstrated statistical significance (p = 0.01). Weekday–weekend midpoint difference was not significantly associated with being overweight/obese in the overall model or in sex-specific analysis (Table 5).

**Table 4:** Associations between weekday and weekend midpoint of sleep and overweight and obesity among adolescents (age 12–17, N = 1,254).

Range of sleep midpoint	Sleep midpoint, quintiles, hour:min									*p* for trend
	Weekday									
	12 a.m.–1:45 a.m.	1:45 a.m. to < 2:10 a.m.		2:10 a.m. to < 2:30 a.m.		2:30 a.m. to < 2:55 a.m.		2:55 a.m.–7 a.m.		
	OR 95% CI	OR 95% CI		OR 95% CI		OR 95% CI		OR 95% CI		
**Overall**										
Base model	Referent	0.99	(0.65, 1.51)	1.26	(0.79, 2.02)	1.21	(0.79, 1.85)	0.95	(0.60, 1.51)	0.76
Model 2	Referent	1.01	(0.66, 1.55)	1.30	(0.80, 2.11)	1.25	(0.81, 1.94)	1.02	(0.63, 1.65)	0.53
Model 3	Referent	1.13	(0.67, 1.91)	1.32	(0.74, 2.37)	1.49	(0.87, 2.56)	1.04	(0.59, 1.85)	0.88
**Female**										
Base model	Referent	0.90	(0.50, 1.61)	0.95	(0.48, 1.84)	0.92	(0.58, 1.94)	1.17	(0.54, 1.81)	0.60
Model 2	Referent	0.86	(0.47, 1.57)	0.96	(0.48, 1.90)	0.96	(0.52, 1.78)	1.38	(0.71, 2.65)	0.31
Model 3	Referent	0.95	(0.50, 1.78)	0.81	(0.39, 1.68)	0.85	(0.44, 1.63)	1.23	(0.61, 2.48)	0.76
**Male**										
Base model	Referent	1.10	(0.60, 2.03)	1.68	(0.85, 3.34)	1.58	(0.85, 2.95)	0.81	(0.41, 1.60)	0.94
Model 2	Referent	1.20	(0.63, 2.27)	1.79	(0.87, 3.68)	1.68	(0.87, 3.25)	0.79	(0.38, 1.65)	0.87
Model 3	Referent	1.26	(0.65, 2.43)	1.76	(0.84, 3.70)	1.69	(0.85, 3.34)	0.73	(0.34, 1.56)	0.64

	Weekend									
Midpoint range of sleep	< 3:30 a.m.	3:30 a.m. to < 4 a.m.		4 a.m. to < 4:30 a.m.		4:30 a.m. to < 5:05 a.m.		≥ 5:05 a.m.		
**Overall**										
Base model	Referent	0.85	(0.55, 1.32)	1.21	(0.80, 1.85)	1.36	(0.92, 1.99)	1.22	(0.80, 1.85)	0.07
Model 2	Referent	0.83	(0.53, 1.30)	1.09	(0.70, 1.68)	1.15	(0.77, 1.71)	1.12	(0.73, 1.72)	0.27
Model 3	Referent	0.76	(0.44, 1.32)	1.28	(0.76, 2.17)	1.35	(0.84, 2.20)	1.16	(0.68, 1.98)	0.53
**Female**										
Base model	Referent	0.94	(0.46, 1.90)	1.95	(1.03, 3.71)	2.17	(1.20, 3.92)	1.78	(0.95, 3.33)	0.01
Model 2	Referent	0.91	(0.44, 1.87)	1.82	(0.93, 3.55)	1.85	(1.00, 3.45)	1.79	(0.93, 3.45)	0.02
Model 3	Referent	0.94	(0.44, 2.01)	1.81	(0.90, 3.63)	1.82	(0.96, 3.47)	1.68	(0.85, 3.33)	0.04
**Male**										
Base model	Referent	0.78	(0.44, 1.40)	0.83	(0.47, 1.47)	0.94	(0.55, 1.58)	0.87	(0.49, 1.55)	0.86
Model 2	Referent	0.73	(0.40, 1.34)	0.71	(0.39, 1.29)	0.73	(0.42, 1.27)	0.69	(0.38, 1.28)	0.28
Model 3	Referent	0.73	(0.39, 1.36)	0.72	(0.39, 1.33)	0.67	(0.38, 1.20)	0.64	(0.34, 1.21)	0.17

Weekday consists of sunday to thursday evenings. Weekend consists of friday and saturday evenings.

Base model adjusted for sex (for overall analysis alone) and age.

Model 2: adjusted for covariates in base model and race, school type, parental marital status, home ownership, parental education.

Model 3: adjusted for covariates in model 2 and moderate-vigorous physical activity (MVPA), teen sedentary time, teen frequency of beneficial foods eaten, teen frequency of detrimental foods eaten, and time spent watching television.

p-for-interaction between sex and sleep timing: 0.45 for weekday and 0.01 for weekend sleep midpoint.

*OR* Odds ratio, *95% CI* 95% confidence interval.

**Table 5:** Associations between Weekday–Weekend differences in sleep Midpoint and overweight and obesity among adolescents (N = 1,254).

Range of weekend–weekday difference in sleep midpoint	Weekend–weekday difference in sleep midpoint, quintiles, hours									
	− 3.58 to 1.0	1.01 to 1.67		1.67 to 2.17		2.18 to 2.75		2.76 to 4.5+		*p* for trend
	OR 95% CI	OR 95% CI		OR 95% CI		OR 95% CI		OR 95% CI		
**Overall**										
Base model	Referent	1.10	(0.70, 1.73)	1.15	(0.75, 1.75)	1.44	(0.97, 2.12)	1.35	(0.89, 2.03)	0.06
Model 2	Referent	0.99	(0.62, 1.57)	1.06	(0.68, 1.64)	1.20	(0.80, 1.81)	1.10	(0.71, 1.71)	0.43
Model 3	Referent	1.29	(0.72, 2.30)	1.35	(0.79, 2.33)	1.47	(0.89, 2.44)	1.40	(0.82, 2.39)	0.45
**Female**										
Base model	Referent	0.85	(0.41, 1.78)	1.08	(0.56, 2.07)	1.57	(0.87, 2.83)	1.39	(0.74, 2.62)	0.07
Model 2	Referent	0.73	(0.34, 1.57)	0.95	(0.48, 1.89)	1.20	(0.64, 2.27)	1.11	(0.56, 2.19)	0.32
Model 3	Referent	0.90	(0.34, 2.38)	1.56	(0.65, 3.72)	1.47	(0.67, 3.23)	1.49	(0.64, 3.47)	0.32
**Male**										
Base model	Referent	1.28	(0.72, 2.3)	1.21	(0.69, 2.12)	1.29	(0.76, 2.18)	1.25	(0.71, 2.19)	0.44
Model 2	Referent	1.24	(0.68, 2.26)	1.12	(0.63, 2.00)	1.07	(0.62, 1.88)	0.96	(0.52, 1.76)	0.79
Model 3	Referent	1.36	(0.94, 4.64)	1.25	(0.61, 2.89)	1.17	(0.75, 3.29)	0.99	(0.57, 2.66)	0.90

Weekday consists of sunday to thursday evenings. Weekend consists of friday and saturday evenings.

Base model adjusted for sex (for overall analysis alone) and age.

Model 2: adjusted for covariates in base model and race, school type, parental marital status, home ownership, parental education.

Model 3: adjusted for covariates in model 2 and moderate-vigorous physical activity (MVPA), teen sedentary time, teen frequency of beneficial foods eaten, teen frequency of detrimental foods eaten, and time spent watching television.

Negative weekend–weekday difference in midpoint values represent later sleep midpoint on the weekdays compared to weekends.

Positive weekend–weekday difference midpoint values represent later sleep midpoint on the weekend compared to weekdays.

p-for-interaction between sex and weekday–weekend difference in sleep duration: 0.25.

*OR* Odds ratio, *95% CI* 95% confidence interval.

## Discussion

In this large study of U.S. adolescents, we found that sleep duration was inversely associated with overweight and obesity, but the associations appeared to be different for males and females, and for weekday and weekend sleep duration. In particular, among males, sleeping 10+ h on weekends was associated with lower odds of overweight and obesity when compared to 8–9 h of sleep on weekends. Among females, greater differences in sleep duration between weekdays and weekends were associated with higher odds of overweight and obesity. We found little evidence supporting a relationship between sleep timing and overweight and obesity in the overall population, although a later weekend midpoint was associated with higher odds of overweight and obesity in females.

Our results of an inverse association between sleep duration and overweight and obesity was largely consistent with findings from previous studies. Several meta-analysis studies that summarized earlier findings reported that individuals with shorter sleep duration had twice the risk of being overweight and obese^5,32^. However, there has been some disagreement about whether this relationship was primarily driven by short or long sleep, or both. Studies have shown that shorter sleep duration is associated with higher body weight in adolescent Hispanic and Caucasian children from the Tucson, Arizona^33^, higher BMI-z score in Korean children and adolescents^34^, and higher BMI in Saudi Arabian boys and girls 10–19 years or age^35^. Others demonstrated associations between longer sleep and lower BMI. For example, in a Korean study, longer sleep on weekdays and weekends was associated with lower BMI in adolescents^36^. Mitchell et al. also demonstrated that when compared to 7.5–8 h, 10 h of sleep was associated with lower odds of overweight and obesity in both male and female adolescents aged 14–18 in suburban Philadelphia^2^. Moreover, a study of over 2000 university students showed that both short and long sleep durations are associated with a higher likelihood of being overweight/obese^37^. Our study suggested that the effects of sleep duration on overweight and obesity differed between males and females and for weekday vs. weekend sleep duration, and these factors should be considered in future studies. In particular, we showed that the protective effects of long weekend sleep were only observed in males, but not in females. Although it is unclear what may have contributed to the observed sex differences, previous studies suggested that both environmental and behavioral factors as well as hormonal changes during adolescence may lead to different sleep patterns between males and females. For example, hormonal factors may be responsible for the more prominent shift toward a later chronotype in boys^13,16^, while emotional and relationship problems may contribute to a higher prevalence of insomnia symptoms in girls^15^. More studies are needed to examine sex-differences in the sleep–obesity relationship in the adolescent population and identify factors and mechanisms that may drive these sex differences. Overall, our study is consistent with the statement issued by American Academy of Sleep Medicine recommending that teenagers 13–18 years of age obtain 8–10 h of sleep to promote optimal health^38^. However, a recent study found that more than 40% of adolescents did not get 7 or more hours of sleep on most nights^39^. Given the growing body of evidence linking sleep deficiency with adverse health outcomes, promoting sufficient sleep in adolescents is an important public health priority.

We found that a larger weekday–weekend difference in sleep duration was associated with overweight and obesity, particularly in females. Moreover, this finding remained after adjusting for average sleep duration, suggesting that sleep duration variability is an independent predictor of BMI status. These findings were consistent with those from several previous studies. In a study of 240 Canadian youth aged 8–17, Jarrin et al. found that longer oversleep on weekends was associated with higher BMI^40^. In addition, higher day-to-day variability in sleep duration has been associated with obesity-promoting diets in Danish children^41^, as well as adolescents from the Penn State Child Cohort study^42^. Moreover, several studies showed that fluctuations in sleep duration may lead to changes in appetite-regulating hormones (e.g. insulin, ghrelin and leptin) that may in turn alter eating behaviors and increase food intake^43–45^. Indeed, weekend “catch-up” sleep has been associated with eating in the absence of hunger, or eating past satiation in response to fatigue, negative affect, or when cued by external circumstances such as the sight or smell of food^46^. However, none of these studies examined sex-specific relationship between sleep variability and obesity, and it is unclear what factors account for the stronger relationship among females in our study, a finding that needs to be confirmed by future studies. In summary, this section of our results suggests that maintaining a regular sleep pattern may contribute to decreased risk of obesity in adolescents.

It has been suggested that sleep timing may also be an important contributor to obesity. Several studies reported that a later sleep timing was associated with overweight and obesity^8–10,13,17,19,27,47–49^. For example, Malone et al. found that midpoint of sleep is positively associated with waist-to-height ratios in adolescents aged 14–17^27^. Additionally, in another study of children 9–16 years of age, later bedtimes and wake up times were associated with higher BMI-z scores^10^. Moreover, some studies also suggested that weekday–weekend differences in the midpoint of sleep, also referred to as social jet lag^13,50^, and may also be a risk factor for overweight and obesity^13,19,27,49^. Previous studies demonstrated that social jetlag was associated with metabolic dysfunction^19^, higher BMI z-scores and waist-to-height ratios^27^ and obesity^13^. In contrast, we found little evidence supporting an association between either sleep timing or weekday–weekend difference in sleep timing and overweight and obesity. However, we found that a later weekend sleep timing was associated with higher odds of overweight and obesity in females. For most adolescents, timing of weekday sleep is largely influenced by school schedule, while the timing of weekend sleep is better aligned with individual preferences. The stronger association between weekend sleep timing and overweight and obesity in females suggest that in this group the internal circadian clock may play a more important role in adolescent weight status. More studies are needed to clarify the relationship between sleep timing and weight status in adolescents.

Our study has several strengths. The study recruited a diverse group of adolescents similar to that of the general U.S. population in terms of sex, income, age, household size, and region. The study also included an even distribution of males and females in a large group of subjects, which allowed us to conduct sex-specific analysis. Furthermore, this study collected data on weekday and weekend sleep separately, which enabled us to examine not only weekday and weekend sleep characteristics separately but also weekday–weekend differences. There are also limitations to our study. First, sleep was self-reported and prone to error and subjective bias. Second, we do not have information on sleep quality, another potentially important predictor of BMI outcomes^37,40,51^. Third, this study was cross-sectional, and therefore it is challenging to establish the direction of the relationships using our data. Finally, although we adjusted for a wide range of covariates as potential confounders, residual confounding may still have an impact on the results.

In conclusion, our results support a role of sleep and its weekly variability in overweight and obesity in adolescents. Our findings have important implications for developing approaches to alleviating overweight and obesity in the United States adolescent population. Moreover, behaviors developed at earlier ages impact later stages of life and health outcomes, suggesting adolescence as a period of life in which interventions can address. We encourage future studies to focus on evaluating the effects of improving sleep hygiene in adolescents to achieve health benefits.
